# The Effect of Simulated Flash-Heat Pasteurization on Immune Components of Human Milk

**DOI:** 10.3390/nu9020178

**Published:** 2017-02-22

**Authors:** Brodie Daniels, Stefan Schmidt, Tracy King, Kiersten Israel-Ballard, Kimberly Amundson Mansen, Anna Coutsoudis

**Affiliations:** 1 Department of Paediatrics and Child Health, School of Clinical Medicine, University of KwaZulu-Natal, Durban 3001, South Africa; Coutsoud@ukzn.ac.za; 2 Discipline of Microbiology, School of Life Sciences, University of KwaZulu-Natal, Pietermaritzburg 3201, South Africa; schmidts@ukzn.ac.za (S.S.); tracyleighking@yahoo.com (T.K.); 3 Maternal, Newborn and Child Health and Nutrition Global Program, PATH, Seattle, WA 98121, USA; kisrael-ballard@path.org (K.I.-B.); kamundson@path.org (K.A.M.)

**Keywords:** human milk, human milk banking, pasteurization

## Abstract

A pasteurization temperature monitoring system has been designed using FoneAstra, a cellphone-based networked sensing system, to monitor simulated flash-heat (FH) pasteurization. This study compared the effect of the FoneAstra FH (F-FH) method with the Sterifeed Holder method currently used by human milk banks on human milk immune components (immunoglobulin A (IgA), lactoferrin activity, lysozyme activity, interleukin (IL)-8 and IL-10). Donor milk samples (*N* = 50) were obtained from a human milk bank, and pasteurized. Concentrations of IgA, IL-8, IL-10, lysozyme activity and lactoferrin activity were compared to their controls using the Student’s *t*-test. Both methods demonstrated no destruction of interleukins. While the Holder method retained all lysozyme activity, the F-FH method only retained 78.4% activity (*p* < 0.0001), and both methods showed a decrease in lactoferrin activity (71.1% Holder vs. 38.6% F-FH; *p* < 0.0001) and a decrease in the retention of total IgA (78.9% Holder vs. 25.2% F-FH; *p* < 0.0001). Despite increased destruction of immune components compared to Holder pasteurization, the benefits of F-FH in terms of its low cost, feasibility, safety and retention of immune components make it a valuable resource in low-income countries for pasteurizing human milk, potentially saving infants’ lives.

## 1. Background

Human breast milk provides infants with nutrition, immune factors, growth factors, digestive enzymes, hormones, and other bioactive factors (such as antibodies (Immunoglobulin M, Immunoglobulin G, and IgA), polyunsaturated fatty acids, soluble receptors (e.g., Cluster of Differentiation 14), cytokines and chemokines, prebiotics, oligosaccharides, immune cells and antibacterial proteins/peptides (e.g., lysozyme and lactoferrin)), and is considered the optimal source of nutrition and immunologic protection for all infants [1,2,3]. Lactoferrin has antimicrobial activity against a large number of bacteria, fungi and even viruses [2]. Lysozyme is able to inhibit the growth of many, mostly Gram-positive bacterial species by disrupting the bacterial cell wall and was shown to inhibit selected yeasts [2,3,4]. Secretory IgA accounts for 90% of total immunoglobulins in milk, and ensures that any antibodies to specific antigens that the mother is exposed to are passed on to the infant [3,4]. Cytokines have been found in significant amounts in breast milk and play an important role in the immune modulation and immune protection of breast milk [5].

Therefore, human milk is especially important for vulnerable infants in resource-limited settings—such as preterm, low-birth-weight, Human Immunodeficiency Virus (HIV)-exposed, or orphaned infants. This group of infants is at increased risk of malnutrition, infectious diseases and mortality, often due to a lack of safe and adequate nutrition. The World Health Organization (WHO) recommends that babies be exclusively breastfed for six months and continue breastfeeding for at least two years thereafter.

Due to its rising infant mortality rate, the South African government was prompted to focus on the underlying causative factors, including low levels of breastfeeding [6]. This was addressed in the update of the country’s infant feeding policy in 2011, when it was declared that South Africa would move to discontinue the free provision of formula milk at hospitals and clinics, and protect, support and promote breastfeeding for all mothers, including those living with HIV [7]. It was also recommended that all hospitals with neonatal intensive care units set up donor human milk banks (also known as Human Milk Banks (HMBs)) to increase access to donor human milk for special cases where the mother’s own milk is not available for these vulnerable neonates, either due to the mother’s inability to breastfeed, maternal death, severe illness or infant abandonment.

Provision of human milk to those infants most in need has been shown to improve infant health and survival as well as reduce the cost burden on the health care system [8,9,10,11,12]. In most countries, HMBs are governed by national or regional guidelines to ensure safe processes are followed [13,14,15]; however, global standards do not exist, although some effort has been made by PATH to consolidate different practices into a Global Implementation Framework [16].

Successful pasteurization of donor human milk involves not only pathogen inactivation but also preservation of milk composition. It is therefore essential that any new pasteurization system achieves the required inactivation of pathogenic microorganisms along with retaining the highest possible level of immune components.

Most HMBs globally utilize the Holder, low-temperature, long-time (LTLT) pasteurization method of heating milk to 62.5 °C for 30 min using commercial pasteurizers which can range in cost from $10,000 to $60,000 each, depending on exchange rates and shipping costs. Low-income countries have a relatively great need for human milk; however, setting up HMBs to meet this need is cost-prohibitive and often small-scale systems are more appropriate at initial stages of implementation. Given this need for low-cost and improved systems, the European Society for Paediatric Gastroenterology Hepatology and Nutrition Committee on Nutrition called for a renewed research focus on the heat treatment of human milk in HMBs [17]. Flash-heat pasteurization (FH) is a simple, manual alternative method of pasteurization which was originally developed specifically for HIV-positive mothers to have a mechanism for heat-treating their own breast milk to render it safe from HIV—prior to the revised WHO guidance for enhanced anti-retroviral use during breastfeeding [18]. At that time, flash-heat was developed to mimic commercial flash pasteurization, a high-temperature short-time heat treatment (HTST) which typically heats samples to 72 °C for 15 s. Although commercial flash pasteurization at 72 °C has mostly been shown to be superior to LTLT methods in terms of the retention of the human milk composition [19,20], the high-tech equipment and high volumes required for this system have not allowed this commercial process to be utilized in human milk banks. 

In the original FH procedure designed for HIV-positive mothers in their homes [21,22,23], 50 mL of milk is placed in an uncovered 450 mL glass food jar procured from local kitchen suppliers, and it is then placed in approximately 450 mL of water in a 1–2 L aluminum pan. The water and milk are heated together over an electric hot plate until the water reaches 100 °C and is at a rolling boil. The jar of breast milk is then immediately removed from the water bath and allowed to cool before being used or it is rapidly cooled in an ice bath and frozen if it is to be stored. FH assessments documented inactivation of HIV, as well as 80% retention of total IgA, and 89% and 43% retention of lactoferrin and lysozyme antibacterial activity, respectively, as well as all of the bacteriostatic activity, compared to raw human milk [11,13]. 

Subsequent to this documentation of safety, FH was also successfully rolled out with HIV-infected mothers in Tanzania who typically heated about 120 mL of milk in each pasteurization process. Microbiological analysis showed that the method was successful in reducing bacterial counts to an acceptable level in all 105 samples collected from mothers [24]. Although not initially developed for the clinical setting with other mothers’ milk, FH was also later proposed as a simple, low-cost pasteurization method in order to provide donor human milk for vulnerable infants in the neonatal intensive care unit (NICU) of one of the district hospitals in South Africa [25]. 

As part of the challenge of scaling up HMBs in South Africa to meet the large need, the possibility of expanding the use of FH pasteurization was considered. It was assumed that based on earlier FH data, which was designed to mimic the commercial HTST method, FH would retain the majority of the protective elements in human milk, and would be potentially superior to Holder pasteurization [20,26,27,28,29,30,31]. 

Rigorous quality control mechanisms, however, were needed to ensure clinical standards for safety and tracking were in place, comparable to global human milk banking models. User guidance, temperature monitoring and tracking were necessary to be sure that appropriate time/temperature curves were documented and archived. Therefore, a low-cost temperature cellphone-based monitoring system, FoneAstra, which was initially designed by the Department of Computer Science and Engineering at the University of Washington (UWCSE) for vaccine cold chains, was adapted, in partnership with PATH and the Human Milk Banking Association of South Africa, for use in monitoring the pasteurization of donor human milk [32]. The strength of the FoneAstra Flash Heating (F-FH) system lies in its simplified use, as well as in the hardware and supporting equipment equating to less than US$1000, which is a fraction of the cost of commercially available human milk pasteurization systems that utilize the Holder method.

The challenge in simulating commercial dairy HTST systems is that typically milk is heated in a high-pressure, continuous flow through fine tubing, allowing for rapid and stringent control of the milk temperature since the milk should remain at 72 °C for only 15 s. Accomplishing this precision and speed for human milk is not feasible due to the cost and lower volumes of human milk compared to bovine systems. As such, automated HTST systems for human milk do not currently exist. Hence the F-FH system was designed to improve the rigor of temperature tracking by monitoring the donor human milk (DHM) heating to a conservative 73 °C and then cooling it as quickly as possible. However, the latent heat from the manual water bath F-FH system would typically result in a peak temperature over 74 °C. 

The purpose of this study was to determine the impact of F-FH on five immunological components of DHM viz. IL-8, IL-10, IgA, lysozyme and lactoferrin. In addition, the study compared the impact of Holder pasteurization (using the Sterifeed Pasteurizer) on these same components.

## 2. Methods 

### 2.1. Sample Processing

**Figure 1:**
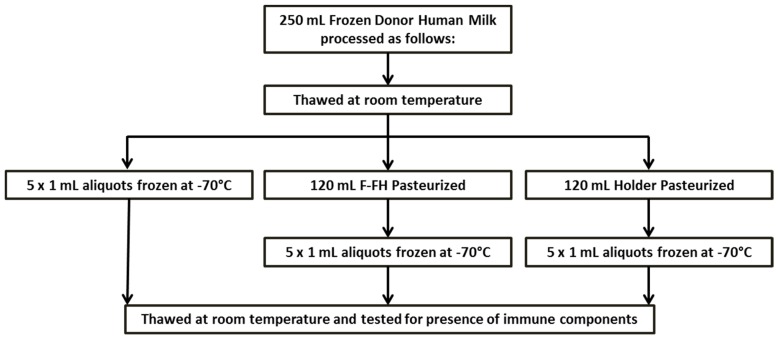
Flow diagram illustrating sample handling and processing. F-FH: FoneAstra Flash Heat.

Fifty samples (250 mL) of frozen anonymous DHM were obtained from a HMB. As per standard regulations, donors were healthy, HIV negative women who were screened for absence of any lifestyle risks and donated breastmilk is stored frozen for a maximum period of three months from date of expression. Donor human milk samples were processed according to Figure 1. Therefore, from each donor human milk sample there were three sets of aliquots: unpasteurized human milk; human milk pasteurized by holder pasteurization using the Sterifeed pasteurizer and human milk pasteurized using the flash-heat (F-FH) method, monitored using the FoneAstra pasteurization system. Unpasteurized samples will be referred to as “Control” samples throughout the remainder of this document, while “F-FH” and “Holder” represent the FoneAstra flash-heated samples and Sterifeed-Holder pasteurized samples, respectively. Cytokine and antibody assays were performed in the Department of Paediatrics and Child Health at the University of KwaZulu-Natal. Lysozyme and lactoferrin activity assays were performed in the Department of Microbiology at the University of KwaZulu-Natal. Samples were assayed between one and 18 months after pasteurization; however, controls and pasteurized samples were always processed together for each variable tested. 

### 2.2. Sample Pasteurization

One glass bottle was pasteurized using the F-FH system according to the 2014 prototype instrument, and the other one was pasteurized in a standard plastic Sterifeed bottle using a commercial Sterifeed pasteurizer (Holder method). The F-FH system involves manual-operation of heating four containers (three with donor milk and one filled with water as a proxy) in a pot of water over an induction stove. Donor information is entered in the phone and upon starting the heating process, the temperature monitor guides the user based on commands displayed on the cellphone screen, signaling when to remove the four containers from the hot water and manually transfer to a cold-water bath for rapid cooling down to 25 °C. This system alerts the user according to the flash-heat temperature, alerting the user at 73 °C to transfer to the water bath. Information collected, including temperature curves, is uploaded to a cloud-based server for remote monitoring. During this study, F-FH temperature thresholds were set at 73 °C to allow conservative heating parameters for piloting in a clinical setting. The Sterifeed human milk pasteurizer is an automated heating/cooling system, heating up to 71 × 120 mL bottles to 62.5 °C for 30 min followed by cooling to 10 °C in the same unit, a process that takes approximately 2 h. 

### 2.3. Cytokine and Antibody Assays

IL-8 and IL-10 were assayed using R&D Systems Quantikine ELISA kits (Minneapolis, MN, USA) as per manufacturer’s instructions. Results for IL-8 and IL-10 are expressed as picograms per milliliter (pg/mL). Total IgA was measured using the Abcam human IgA ELISA kit (Cambridge, UK) as per manufacturer’s instructions. Results for IgA are expressed as micrograms per milliliter (µg/mL).

Briefly, all samples were assayed in duplicate. Samples were thawed completely and vortexed thoroughly before being assayed. Controls and their two pasteurized samples were always run simultaneously on the same ELISA plate. Samples were diluted 1:20,000 for the IgA assays, while no dilution of samples was necessary for the cytokine assays. 

### 2.4. Lactoferrin Bacteriostatic Activity Assay

Kundrat agar (Sigma-Aldrich, Johannesburg, South Africa) was prepared according to the manufacturer’s recommendations. After autoclaving at 121 °C for 15 min, the agar was cooled in a water bath to 55 °C, 2 mL of *Geobacillus stearothermophilus* endospore suspension (Merck, Darmstadt, Germany) was added to 200 mL molten agar to a final endospore concentration of approximately 10^6^ per mL, followed by mixing and pouring 15 mL of agar into sterile Petri dishes. After solidification, 5 mm sample wells (three per 90 mm Petri dish) were created using a sterile Pasteur pipette. Each human milk sample was assayed in duplicate by filling wells with 25 μL of human milk sample, followed by incubation at 42 °C for 20 h. Results were established by measuring zones of endospore germination inhibition after incubation using a digital Vernier calliper (Marshal Tools, Durban, South Africa). These were converted to mg of lactoferrin per mL of human milk values by establishing a calibration curve using authentic human lactoferrin as a standard (Sigma-Aldrich, Johannesburg, South Africa).

### 2.5. Lysozyme Activity Assay

Lysoplates were prepared as described by Osserman and Lawlor [33] and Jenzano and Lundblad [34], by adding 50 mg heat-inactivated *M. lysodeikticus* cells (Sigma-Aldrich, Johannesburg, South Africa) to 100 mL molten (60–70 °C) 1% agarose (Whitehead Scientific, Cape Town, South Africa) in 66 mM phosphate buffer (pH 6.6). Aseptically, 15 mL of *M. lysodeikticus* containing molten agarose was added to separate Petri dishes to a depth of 4 mm. After solidification, 5 mm sample wells (three per 90 mm Petri dish) were cut with a sterile Pasteur pipette. Each breast milk sample was assayed in duplicate by the lysoplate technique. Sample wells were filled with 25 μL of appropriately diluted (using the above buffer) breast milk sample followed by incubation of the plates at 37 °C for 20 h. Results were established by initially measuring zones of lysis after incubation using a digital Vernier calliper (Marshal Tools, Durban, South Africa). These were then converted to mg of lysozyme per mL of breast milk using human lysozyme (Sigma-Aldrich, Johannesburg, South Africa) as a standard to establish a calibration curve.

### 2.6. Ethical Considerations

Ethical approval for the study was granted by the Biomedical Research Ethics Committee of the University of KwaZulu-Natal (Approval date: 14 April 2014; approval code: BE 114/14).

## 3. Statistics

Immune factor concentrations are not normally distributed; therefore, a nonparametric test for paired samples was used to test the significance of these data. Differences between group pairs were analyzed with a Student’s *t*-test, using a Wilcoxon signed rank post-test. When comparing the percentage retention of immune factors, a Student’s *t*-test with a Mann-Whitney post-test was used. Outliers were identified and removed from the analysis using the Rout method, establishing a maximum false discovery rate (FDR) of 1% (*Q* = 1). All analyses were performed using GraphPad Prism version 7 (GraphPad Software, San Diego, CA, USA). Data in bar graphs are presented as means ± Standard Deviation (SD). Findings were assumed statistically significant at *p* < 0.05. The value of the controls was considered to be 100%; therefore, the concentration of cytokines and antibody and the amount of lysozyme and lactoferrin activity were expressed as a percentage of the control sample. 

## 4. Results

Of the 50 samples, 48% (24 of 50 samples) were from mothers with preterm deliveries. Donors were mostly Caucasian (76%), with 10% Indian, 12% African and 2% mixed race descent.

Table 1 shows the mean concentrations of each component (±SD), in addition to the mean percentage retention (±SD). The results show that both the F-FH and Holder samples were significantly different to the control samples, except for IL-10. 

Neither of the methods caused a substantial decrease in IL-10 (Table 1). However, both methods showed a slight but significant increase in IL-8.

The Holder method showed a small but significant increase in lysozyme retention, while causing a significant decrease in the retention of lactoferrin (71.1% retention) and IgA (78.9% retention). The F-FH method also showed a significant reduction in lactoferrin (38.6% retention) and IgA (only 25.2% retained). However, the lysozyme was less affected than the other two components with 78.4% retention.

**Figure 2:**
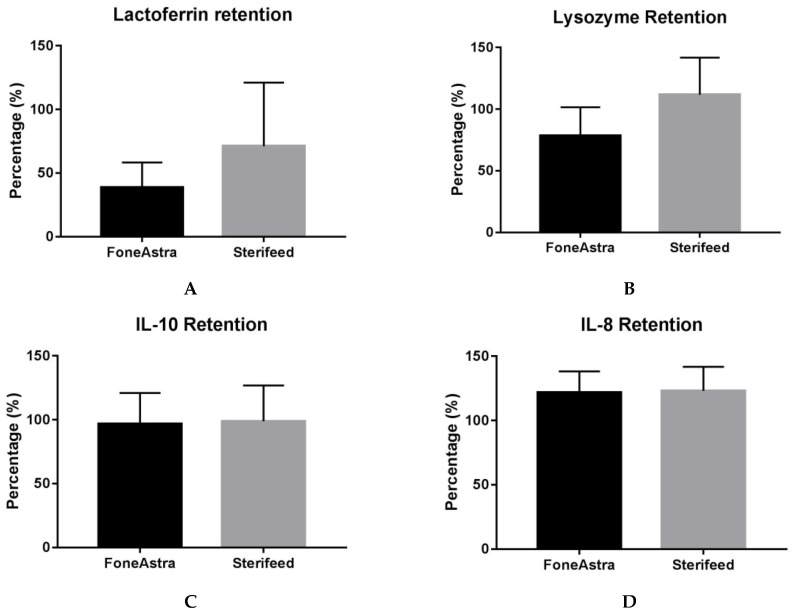

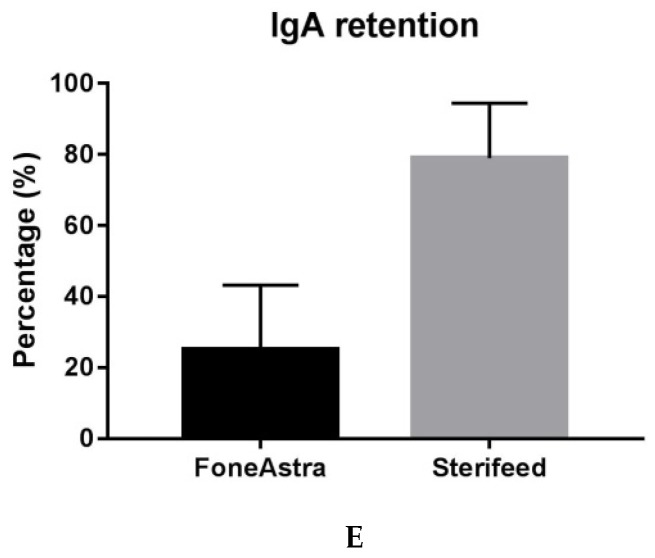
Effect of F-FH and Holder pasteurization on the retention of immune components expressed as percentage retention (percentage of control). (**A**) Lactoferrin; (**B**) Lysozyme; (**C**) IL-10; (**D**) IL-8 and (**E**) IgA. Bars represent means ± SD. * <0.0001. F-FH: FoneAstra Flash Heated; IL: Interleukin; SD: standard deviation; IgA: Immunoglobulin A.

When the two methods are compared (Figure 2), a significant difference is seen with regard to IgA retention (25.2% in F-FH vs. 78.9% in Holder), lysozyme retention (78.4% in F-FH vs. 100% in Holder) and lactoferrin retention (38.6% in F-FH vs. 71.1% in Holder).

## 5. Discussion

This study found variations in the impact on human milk immunological properties, based on the pasteurization method and the corresponding temperature curves. The lack of negative impact on the cytokines studied was expected [35,36,37] and both methods of pasteurization showed a slight increase in cytokines, but no decrease. The effect of Holder pasteurization on milk cytokines appears to be cytokine-dependent [38], while the increase in IL-8 has previously been noted with Holder pasteurization [37]. This could be explained by the heat from pasteurization causing a release of unbound IL-8 protein, making IL-8 more readily able to bind the IL-8 antibody in the ELISA assay [37]. The percentage of retention of lysozyme with the FoneAstra pasteurization (74.8%) was within acceptable levels when compared to other methods of pasteurization, although it was surprising that with the Holder method there was an increase in lysozyme activity. This finding agrees with previous data showing lysozyme activity to be minimally affected by the Holder method [20,26,28] or even increased [28] as reported in this study.

Both pasteurization methods tested resulted in a decrease in lactoferrin and IgA. Human milk subjected to Holder pasteurization resulted in 71.1% lactoferrin retention compared to 38.6% with F-FH. IgA retention was 78.9% when subjected to Holder pasteurization, compared to 25.2% with F-FH. The level of destruction of the immune components IgA and lactoferrin with Holder pasteurization reported in this study is comparable to that reported in the literature [27,30,31]. Previous studies have assessed the impact of various methods of pasteurization on human milk immune components, although little work has examined the impact of flash-heating. Our F-FH results differ from the single study exploring the impact from the initial FH system. Previous data showed higher levels of retention of IgA (80%) and lactoferrin antibacterial activity (89%) but lower levels of retention of lysozyme antibacterial activity (43%) (Table 2) [23,39]. 

**Figure 3:**
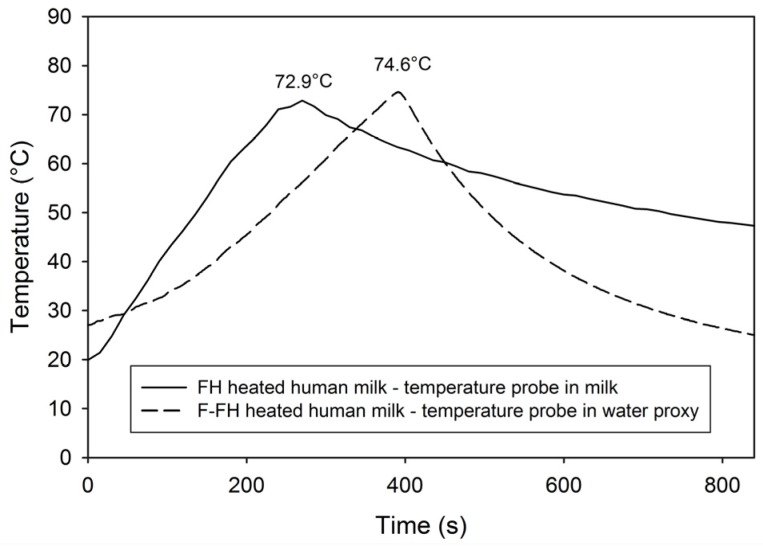
Time-temperature curves of the original FH study compared to the F-FH used in this study. FH: Flash heated; F-FH: FoneAstra Flash heated.

Although the previous FH study did not compare effects to another pasteurization method, the disparity in these findings was surprising and suggests that even small differences in temperature thresholds and methodologies are critical. Figure 3 shows the time-temperature curves of the original FH study overlayed with a typical curve from this F-FH study. In the original FH study, the temperature probe was placed in a milk sample, and the peak temperature of the milk reached was 72.9 °C, whereas in the current study the peak temperature reached was most likely higher. A significant limitation of the current study is the lack of milk temperature data to document this; the exact temperature in the milk was not monitored directly—instead, the temperature probe in F-FH was placed in a proxy water bottle where the peak temperatures reached were typically between 74–75 °C. The peak temperature in the milk, resulting from the latent increase in heating even after being removed from the water bath, was therefore likely about 74 °C or potentially higher. After samples were collected for this study and these higher peak temperatures were observed, the FoneAstra temperature threshold was subsequently reduced from 73 °C to 72 °C. Previous studies evaluating the effect of varying the pasteurization temperature showed that even small temperature increments can significantly compromise some immune components [20,21,41,45]. Additionally, other factors that differed between the original FH system and the F-FH system, such as the volume of milk pasteurized, the bottle size used and the heat source, would likely have contributed to the differences seen between the outcomes of the two studies.

The critical finding of this research is documenting the negligible margin of tolerability of temperature variation that is allowable during the pasteurization of human milk. This is substantiated by the work of Naicker et al. [46] testing the impact of FH without temperature monitoring with F-FH, as no human milk samples subjected to F-FH (probably with higher peak temperatures) showed bacterial growth after pasteurization, whereas 1% of human milk samples treated with FH without temperature monitoring (probably lower peak temperatures) did show bacterial contamination. 

Although developed for the dairy industry, thermal pasteurization has been used as the primary method of human milk treatment by human milk banks. However, optimal heating and cooling profiles specifically for human milk have not been established which would both maximize the retention of unique human milk protective elements and inactivate pathogens, resulting in the highest quality DHM for at-risk neonates. As a result, proposed innovations in human milk pasteurization, including FH, lack clear guidance for systems development. Research is needed to establish the optimal pasteurization parameters for maximum immune component retention and pathogen inactivation in DHM.

This study has provided valuable information on good indicators to use to test optimum temperatures for pasteurization. Based on these findings we suggest that studies could simply choose two indicators viz. those most severely affected by small changes in temperature, such as the retention of IgA and the antibacterial activity of lactoferrin.

## 6. Limitations

This study specifically chose to test two pasteurization systems in exactly the same way that they are conducted in routine practice. This is therefore a limitation in the F-FH system, since temperature monitoring of the process involves placing the temperature probe in a bottle of water and monitoring the heating curve in water as a proxy for human milk. Therefore, accurate heating curves for F-FH human milk are not available. Additionally, the location of the probe in the liquid can change temperature readings substantially; these changes in location were not controlled for in the Holder and F-FH methods and, subsequently, accuracy of the temperature data could be affected. Optimal temperature curves delineating specific time/temperature parameters for human milk pasteurization are yet to be defined and this remains a critical gap in the field. As per normal procedures in human milk banking, the donor milk samples were subjected to several freeze/thaw cycles. Additionally, as part of standard laboratory procedures, the samples taken for laboratory assay also had to undergo a freeze/thaw cycle. While this likely had some effect on the protein structure of the immune components, all samples (including the controls) were subjected to the same freeze/thaw cycles, and therefore any changes seen were probably due to the pasteurization process. The long-term storage of frozen samples before assaying for immune components could also have affected the results seen. However, controls and pasteurized samples were always processed simultaneously; therefore, any possible destruction due to long-term frozen storage would be uniform across the groups. Additionally, a study has previously shown that long-term storage of milk samples at −70 °C did not result in a significant decrease in proteins, secretory IgA or lactoferrin [47].

## 7. Conclusions

Ensuring that donor human milk is safe from pathogens and provides immune protection to vulnerable infants is a complex balance between heating milk high enough to destroy bacteria but not too high to reduce immune components substantially. Our finding that slight changes in heating methodology result in significant changes in the retention of human milk components is an important one. Simple pasteurization devices are needed to ensure the feasibility of setting up and running small-scale human milk banks for increased access to donor human milk. As such, F-FH remains a valuable resource for providing safe human milk for vulnerable infants in resource-limited settings and continuing the optimization of the device is important to align with safety data. Research and resources are needed in this area to improve the quality of donor human milk and to ensure maximum protection is provided to recipient infants. Naicker et al. reported the safety of FoneAstra by reducing the number of bacteria present to below the limit of detection [46]. Despite the partial destruction of IgA and lysozyme and lactoferrin activity reported here, some benefit from these immune components will still be available for infants, as none of these components were completely destroyed. The benefit of receiving pasteurized human milk compared to formula outweighs the partial destruction of these immune components. Our simultaneous work (reported separately) on the effect of pasteurization on human milk oligosaccharides showed F-FH to retain oligosaccharides. This is important as oligosaccharides make up a large portion of human milk, and apart from their nutritional value they have recently been demonstrated to have a wide variety of immune effects on infants, including blocking bacterial infection in the gut and acting as a prebiotic for healthy gut microbiota to flourish [2,3].

In conclusion, despite two immune components (IgA and lactoferrin) being negatively impacted, a further four components (IL-10, IL-8, lysozyme, and oligosaccharides) were unaffected or minimally affected. On a balance of scale of weighing the benefits of low cost, feasibility, safety and minimum destruction of immune components, F-FH remains a valuable resource in low-income countries for pasteurizing human milk, potentially saving infants’ lives. 

## Figures and Tables

**Table 1:** Concentrations of immune components and percentage retention of immune components with different pasteurization methods. Values listed are means (± standard deviations).

	Control (±SD)	Holder (±SD)	F-FH (±SD)	Holder Retention (%) (±SD)	F-FH Retention (%) (±SD)
**Lactoferrin (mg/mL)**	0.061 (0.070)	0.033 (0.039) *	0.023 (0.024)^*^	71.1 (50.0)	38.6 (19.7) *
**Lysozyme (mg/mL)**	0.35 (0.23)	0.37 (0.23) **	0.26 (0.15) *	100 (30.2) ^#^	78.4 (23.2) *
**IL-10 (pg/mL)**	10.77 (7.86)	10.49 (7.81)	10.48 (7.39)	98.8 (28.0)	96.7 (24.3)
**IL-8 (pg/mL)**	186 (145.2)	233.2 (177) *	222.3 (166.5) *	100.0 (18.7) ^#^	100.0 ^#^ (16.4)
**IgAi (µg/mL)**	2800 (2416)	2340 (2159) *	567.6 (740.6) *	78.9 (15.6)	25.2 (18.1) *

* *p* < 0.0001; ** *p* < 0.05; ^#^: where retention was greater than 100% in terms of clinical significance the value was rounded down to 100%. F-FH: FoneAstra Flash Heated; SD: standard deviation; IgA: immunoglobulin A; IL: Interleukin.

**Table 2:** Percentage retention of immune components analyzed in this study according to pasteurization methods compared to previous reported studies.

	Holder-Previous Studies	Holder This Study	FH Previous Study	F-FH This Study
**Lactoferrin antibacterial activity**	15%–100% [20,28,40,41]	71%	89%	39%
**Lysozyme antibacterial activity**	39.4%–106% [20,26,28,29,30,40,41,42]	100%	43%	78%
**IL-10**	Decrease or no effect [35,36,37]	99%	n/a	97%
**IL-8**	Increase [35,36,37]	100%	n/a	100%
**IgA**	43%–100% [20,26,28,29,30,31,41,42,43,44]	79%	80%	25%

FH: Flash heated; F-FH: FoneAstra Flash heated; IL: Interleukin; n/a: not applicable.
